# How does Community-Led Total Sanitation (CLTS) affect latrine ownership? A quantitative case study from Mozambique

**DOI:** 10.1186/s12889-018-5287-y

**Published:** 2018-03-21

**Authors:** Miriam Harter, Sebastian Mosch, Hans-Joachim Mosler

**Affiliations:** Eawag: Swiss Federal Institute of Aquatic Science and Technology, Überlandstrasse 133, 8600 Dübendorf, Switzerland

**Keywords:** RANAS, CLTS, Behavior change, Psychosocial determinants, Social context

## Abstract

**Background:**

Community-led total sanitation (CLTS) is a widely used, community-based approach to tackle open defecation and its health-related problems. Although CLTS has been shown to be successful in previous studies, little is known about how CLTS works. We used a cross-sectional case study to identify personal, physical, and social context factors and psychosocial determinants from the Risks, Attitudes, Norms, Abilities, and Self-Regulation (RANAS) model of behavior change, which are crucial for latrine ownership and analyze how participation in CLTS is associated with those determinants.

**Methods:**

Structured interviews were conducted with 640 households in 26 communities, where CLTS had been completed before and compared to 6 control communities, all located in northern Mozambique in 2015. To identify crucial factors for latrine ownership, logistic regression analysis were conducted and mediation analysis were used to analyse the relationship between CLTS participation and latrine ownership mediated by factors identified by the logistic regression analyses.

**Results:**

Mediation analysis reveal that the relationship of CLTS participation with probability of owning a latrine is mediated by social context factors and psychosocial determinants. Data analysis reveal that the probability of building a latrine depends on existing social context factors within the village, the behavior of others in the community, the (dis)approval of others of latrine ownership, personal self-confidence in latrine building, and a precise communication of the benefits of latrine ownership during a CLTS triggering event.

**Conclusions:**

By including activities to focus on the mentioned factors, CLTS could be improved. Exemplary adaptations are discussed.

**Electronic supplementary material:**

The online version of this article (10.1186/s12889-018-5287-y) contains supplementary material, which is available to authorized users.

## Background

In recent years, one form of intervention to reduce or eliminate open defecation has gained attention worldwide: community-led total sanitation (CLTS). This set of community-based activities was first introduced by Kamal Kar in Bangladesh in 2000 [1]. It is designed to engage individuals in action to eliminate open defecation and recognize health as a common good, worth fighting for as a whole community. It has the potential to replace a top-down approach to subsidizing toilet facilities. The good news is that CLTS is successful in evoking change: people in a variety of cultural settings have started building latrines after participating in CLTS [2–6], for example in Mali [7] and Tanzania [8]. But in the case of Mozambique, the results of CLTS are not encouraging so far: Pendly et al. [9] reported 24% of communities failed to adopt latrine construction after CLTS implementation and Godfrey et al. [10] reported 29% of communities losing the gained status of an open defecation free community.

The results show that many people decide to construct latrines after participating in CLTS, but a high proportion still does not. So far, the conditions under which and how CLTS leads people to decide to construct a latrine remain unclear. A thorough understanding of how CLTS motivates people to take this decision could help improve its effectiveness.

To understand the decision to construct a latrine, we use the risks, attitudes, norms, abilities, and self-regulation (RANAS) model of behavior change [11]. The RANAS approach to systematic behavior change contains five blocks of psychosocial factors. The risk factors block comprises factors that represent a person’s understanding and awareness of the health risk posed by open defecation. These factors are perceived vulnerability, a person’s subjective perception of his or her risk of contracting diarrhea due to open defecation, and perceived severity, a person’s perception of the seriousness of the consequences of contracting diarrhea. Additionally, people should have an understanding (factual knowledge) of how they could be affected by diarrhea through open defecation. Attitude factors are a person’s positive or negative stance towards the construction of a latrine. They comprise cost/benefit (e.g., how time consuming constructing is) and affective (e.g., being proud of having a latrine) evaluations. Norm factors represent the perceived social pressure to construct a latrine. They comprise the descriptive norm (perceptions of latrine construction by others) and the injunctive norm (perceptions of whether latrine construction is typically approved or disapproved of by important others). The ability factors denote a person’s confidence in her or his ability to construct a latrine, beliefs in her or his capacity to organize and execute the course of actions required, and action knowledge, knowing how to construct a latrine. Self-regulation factors represent a person’s attempts to plan and self-monitor and to manage conflicting goals and distracting cues when constructing a latrine; both planning and measures that keep the plan on track are needed to build and maintain a latrine.

Additionally, context factors may play a role in the decision to construct a latrine. To conceptualize conditions in the social, physical, and personal contexts that might influence latrine construction, we refer to the theory of triadic influence [12]. The social context is constituted by culture and social relations, laws and policies, economic conditions (e.g., income, household size), and the information environment (e.g. a CLTS intervention). The official handbook for CLTS states that the program “concentrates on the whole community rather than on individual behaviors” [1], and its aim is to raise awareness of the “collective benefit from stopping open defecation” (page 8). Therefore, the interaction of community members towards a common goal has to be taken into consideration, including such concepts like social capital that include the felt trust of individuals to each other, cooperation between community members, communication, and social cohesion of individuals [13]. Furthermore, how community members feel identified with each other and their social group (as reflected in the concept of social identity) is important to understand the motives behind an ongoing commitment and action towards a common goal, since it also influences, for example, the strength of in-group ties [14]. Defecating in the open can be seen as a social dilemma as well [15]: Even if only one household does not use a toilet, the health of everybody else will be affected. However, this household saves both money and the effort of building a latrine. Whether social trust in building latrines cooperatively is strong or weak and whether individual households assess the effort required as high or low may also influence latrine construction.

The physical context consists of the natural and built environment. Both soil conditions and reasons for the collapse of latrines have to be considered. For instance, whether the soil is loose or firm makes building a latrine more or less easy, and frequent collapses of latrines due to flooding make maintenance and rebuilding laborious.

Finally, the personal context is formed by socio-demographic factors such as age, sex, education, wealth, religion, general abilities (e.g. reading and writing skills), and the physical and mental health of the person. Persons with greater ability to construct a latrine, because they are young, well educated, and physically and mentally strong, may well be more suited to latrine construction.

### The present study

his study investigated the relationships between latrine ownership and psychosocial factors and context factors by conducting a cross-sectional survey in Mozambique. It analyzed how latrine owners differ from non-owners in these factors.

In 2012, Pathfinder International started implementing CLTS in their target regions. The research project involved communities in which CLTS had been completed at least 8 months before the survey (November 2014) and control communities in which CLTS had not yet been implemented. The intervention followed the recommendations in the handbook for CLTS [1]. CLTS consists of three phases: during pre-triggering, CLTS facilitators evaluate the physical and social contexts and current conditions and practices so that they can adapt CLTS to existing circumstances. During the CLTS triggering event, a range of techniques (such as transect walks or open defecation mapping) are used to encourage collective action towards an open-defecation-free environment. This is then supported and monitored during the post-triggering phase.

This paper addresses the following research questions:

1. How successful was CLTS in Mozambique: Do more households that participated in CLTS own latrines than households that did not participate in CLTS?
2. Which psychosocial factors and which context factors are positively or negatively associated with latrine ownership?
3. Which psychosocial factors and which context factors mediate the association between CLTS participation and latrine ownership?

## Methods

### Research area and participants

Data were collected in rural Mozambique in the northern region of Nampula. Communities were selected in which SCIP Nampula (a program lead by Pathfinder International and partners, funded by USAID) had realized CLTS in the past 8 months. Second selection criterion was a size of more than 20 households. Out of all suitable communities, 26 were randomly selected. Additionally 6 communities were randomly selected, where CLTS had not been realized so far to form a control group. A team of 10 data collectors was trained on the questionnaire before the survey. The training included underlying psychological concepts and interviewing guidelines, roleplays on the questionnaire, and detailed discussions of ethical considerations. The team was accompanied by two study managers and one field supervisor. In each community, a random sample of 20 households was selected using a modified random route method based on Hoffmeyer-Zlotnik [16]. The structure of each community was outlined with community members and data collectors were assigned randomly to different sections of the community. Reached there, the data collectors randomly selected every third household in their section. The target respondent was the person chiefly responsible for child care and food preparation; this person was thought the most likely to have the deepest insight into the defecation habits of all the family members and the overall hygiene conditions in the household. In case consent to participate was not given (22 households) or the person encountered did not fit the inclusion criteria or no one was at home (292 households), the data collectors were instructed to continue with the next following house until the sample of 20 households per community was reached. The interview lasted approximately one hour.

### Sample

The households being part of the survey are spread over four districts, with 6.8% in Meconta, 29.9% in Angoche, 32.4% in Monapo, and 30.9% in Mogovolas. The total sample size was 640 households in 32 communities. Missing data led to 37 cases being excluded from the analysis. The sample for data analysis comprised 603 cases.

### Questionnaire and measures

A structured questionnaire was constructed based on the RANAS model [11]. It contained questions about the socio-demographic characteristics of the household, the frequency of latrine use and open defecation of all household members using the Safe San Index [17], and psychosocial factors potentially steering latrine use, open defecation, and latrine construction. Some questions were incorporated to assess the CLTS interventions and subsidy policy. Finally, items assessing social capital, social cohesion, and social dilemma were also included to gain an insight into the social dynamics of the communities. The items were answered using 5-point scales for unipolar items and 7-point scales for bipolar questions; all answers were self-reported. A short spot-check observation of hygiene conditions in the household and the latrine was appended to the end of the questionnaire. The questionnaire was first drafted in English, then translated into Portuguese and discussed with the data collectors in the local language, Makhuwa. The questionnaire was pretested in 20 interviews under real conditions and adapted where necessary.

The CLTS-related information received was used to differentiate between four different groups (Table 1) to show differences in latrine ownership: A) households which participated in CLTS (=participation); B) households which did not attend the CLTS intervention realized in their community personally but received CLTS-related information indirectly from relatives, friends, and neighbors (=no participation/ information received); C) households living in communities that underwent a CLTS triggering event but did not receive any CLTS-related information (=no participation/ no information received) and finally D) households living in the control communities, where CLTS was not performed (=no CLTS village). Furthermore, a dichotomous primary independent variable, CLTS intervention, was created from the four groups to conduct a multiple mediation analysis. The first set of CLTS receivers comprised groups A and B; the second set of CLTS non-receivers was formed by combining groups C and D. CLTS receivers were coded ‘1’, and non-receivers were coded ‘0’.

**Table 1:** Quantity of CLTS-related information

Group label	Quantity of CLTS information received	CLTS intervention
A	Participation	Receivers
B	No participation/information received	Receivers
C	No participation/no information received	Non-receivers
D	No CLTS village	Non-receivers

Latrine ownership was further used as dichotomous dependent variable in four logistic regression models and in multiple mediation analysis. For the status of ownership of a household latrine, self-reported and observed information was combined.

Furthermore, context factors as displayed in Table 2 were used as independent variables in logistic regression analysis.

**Table 2:** Personal, Physical, and Social Context Factors

Personal context	Physical context	Social context
Age	Risk of flooding	Social dilemma
Relationship status	Soil conditions	Social capital
Years at school		Social identity
Ability to read/ write		Social cohesion
Religion		
Household size		
Average monthly income/family		

Self-reported relationship status and ability to read and write are dichotomous items that distinguish people in a relationship from people not in a relationship and individuals who are able to read and write from those not able to read or write. Religion of respondents is categorized as Muslim, Catholic, and tribal or other religions. Soil conditions are classified by respondents as sandy, clayey, or rocky. Continuous scales are used for age, years at school, household size, average monthly income, risk of flooding, and all the social context factors, based all on self-reported data (details of the measurements of these factors are displayed in Additional file 1). Moreover, psychosocial determinants of the RANAS model were assessed using questions that framed latrine construction as a target behavior (for details of the questions see Additional file 2).

### Data analysis procedure

To answer the first research question, we compared four different groups in their latrine ownership status. The four groups are differentiated by the quantity of CLTS-related information they received, as described above (Table 1). The four groups are A) participation, B) no participation/ information received; C) no participation/ no information received and D) no CLTS village.

To answer the second research question, four logistic regression models were performed to identify contextual and psychosocial factors which promise to be relevantly associated with latrine ownership. The first model tested for personal and physical context factors, the second tested for social context factors, and the third tested for psychosocial factors. In the fourth logistic regression model, significant factors from the first three models were included to determine which factors remain crucially related with latrine ownership.

To answer the third research question, a multiple mediation analysis was conducted to identify the social context factors and psychosocial factors that mediate the association of CLTS with latrine ownership. To do this, all statistically significant social context and psychosocial factors from the fourth logistic regression model were included as mediators in this analysis. The binary outcome variable differs between people with and without latrine, and the dichotomous independent variable discriminates between CLTS receivers and non-receivers. The multiple mediation analysis was performed using PROCESS for SPSS [18]. All data analysis was carried out using SPSS version 22.

## Results

The sample consisted of 99.5% female respondents; the mean age was 34 years (SD = 13.6). Of all the respondents, 83.7% reported that they were in a relationship. The respondents had attended school for 2.3 years on average (SD = 2.5); 15.1% were able to read and write. Some 43.2% were Muslims, 49.5% were Catholic and 7.3% belonged to tribal religions or others. The average household contained 5 members (SD = 1.9), and the mean income was MZN 1406.20 (approx. USD 17.80, exchange rate 03.10.2016) per household (SD = USD 30) per month. Of all respondents, 60.6% indicated that their village is not at all subject to flooding, 17.3% reported their village as somewhat at risk for flooding, 8.5% said rather at risk, 10.3% estimated the risk for flooding as quite high, and 3.3% indicated that their village is very much subject to flooding. Moreover, 39.4% of the interviewees reported that they lived in an area with sandy soil conditions, 50.9% lived in areas with clayey soil, and 9.7% in areas with rocky soil conditions. In the total sample 61.4% (*n* = 370) of the respondents reported to be in possession of an own latrine. Of those having an own latrine, 94.1% (*n* = 348) reported using it exclusively. Other 5.4% (*n* = 20) reported to use the latrine but practice open defecation as well and 0.5% (n = 2) only defecated in the open besides owning a latrine. To answer the first research question (Do more households that participated in CLTS own latrines than households not having participated CLTS?) we compared frequencies of latrine ownership for four different groups in the sample (Table 1).

**Fig. 1:**
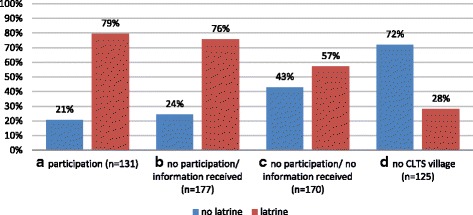
Differences in latrine ownership status related to the extent of which people received CLTS-related information. **a**) households which participated in CLTS (=participation), *n* = 131; **b**) households which did not attend the CLTS intervention in their community personally but received CLTS-related information indirectly from relatives, friends, and neighbors (=no participation/ information received), *n* = 177; **c**) households in communities that underwent a CLTS triggering event but which did not receive any CLTS-related information (=no participation/ no information received), *n* = 170 and finally **d**) households in control communities where CLTS was not performed (=no CLTS village), *n* = 125

As can be seen in Fig. 1 the proportion of people owning a latrine is increasing with increasing extend of CLTS-related information and highest in the group of CLTS participation (79%).

To answer the second research question (Which structural, personal and social context factors and psychosocial factors are positively or negatively associated with latrine ownership?) we conducted four logistic regression analyses with the likelihood that participants own a latrine as dependent variable. Here we display only the fourth regression model; the others can be found in the Supporting Information. The linearity of the continuous variables of all regressions was assessed using the Box-Tidwell procedure [19]. Based on this assessment, all continuous independent variables were found to be linearly related to the logit of the dependent variable.

The first regression model estimates the influence of personal and physical context factors on the probability that people own a latrine (Additional file 3). The logistic regression model was statistically significant, χ^2^(9) = 107.174, *p* < .0005. The model explained 24.2% (Nagelkerke *R*^*2*^) of the variance in latrine ownership and correctly classified 73.3% of cases. Of all nine predictor variables, three context factors remained statistically significant: relationship status, years at school, and risk of flooding (Additional file 1). People in a relationship were 2.35 times more likely to own a latrine than those who were not. More years at school and living in villages with lower risk of flooding was also associated with latrine ownership.

The second regression model estimates the association between social context factors and latrine ownership (Additional file 3). The logistic regression model was statistically significant, χ^2^(10) = 126.998, *p* < .0005. The model explained 25.9% (Nagelkerke *R*^*2*^) of the variance in latrine ownership and correctly classified 71% of cases. Of all ten social context factors, four emerged as statistically significant and are associated with latrine ownership (Additional file 2): social dilemma, solidarity (social capital), trust (social capital), and social cohesion and inclusion (social capital). This indicates that people that perceive their community as having a higher collective ambition to reduce open defecation, greater solidarity within the village, higher trust between the residents, and a stronger sense of cohesion and inclusion within the village are all associated with the individual likelihood of owning a latrine.

The third regression model determines psychosocial factors and their connection with latrine ownership (Additional file 3). The logistic regression model was statistically significant, χ^2^(21) = 426.853, *p* < .0005. The model explained 70.1% (Nagelkerke *R*^*2*^) of the variance in latrine ownership and correctly classified 88.1% of cases. In total, seven psychosocial factors are significant: vulnerability, feelings, beliefs about costs and benefits, others’ behavior, others’ (dis)approval, confidence in recovery, and communication. This means that feeling less vulnerable to becoming infected with diarrhea and feeling not more respected by community members because of owning a latrine is associated with latrine ownership. Beyond that, lower cost expectations about latrine construction, a community with higher rates of latrine ownership, and higher approval of latrine building from others who are personally important to the respondent are also associated with latrine ownership. In addition, having more confidence in being able to rebuild a damaged latrine and talking more frequently about latrine-related topics is associated with latrine ownership.

In the fourth and final regression model, significant context and psychosocial factors from the first, second, and third models were combined to determine the factors associated with latrine ownership (see Table 3). The logistic regression model was statistically significant, χ^2^(15) = 468.192, *p* < .0005. The model explained 74.3% (Nagelkerke *R*^*2*^) of the variance in latrine ownership and correctly classified 87.6% of cases. In combining all factors, relationship status from the personal and physical context factor block lost their significant relationship with the likelihood of latrine ownership, as did social dilemma and social capital (trust and solidarity) from the social context factors block. There remain significant associations between latrine ownership and years at school, risk of flooding, and social capital (social cohesion and inclusion). Spending more years at school, living in villages which are at lower risk for flooding, and living in villages with a stronger sense of social cohesion and inclusion are all associated with latrine ownership. However, all psychosocial factors except communication retained their significant associations in distinguishing between latrine owners and non-owners, and their effects pointed in the same direction as in regression model 3.

**Table 3:** Predictors of Latrine Ownership in Logistic Regression Analysis

Model	*B*	*SE*	Wald X^2^ (1)	OR	95% CI
Model 4: significant context and RANAS factors from model 1 + 2 + 3					
Context factors					
Relationship status^a^	.545	.388	1.969	.58	.27, 1.24
Years at school	.188	.070	7.247**	1.21	1.05, 1.39
Risk of flooding	−.351	.128	7.546**	.70	.55, .90
Social dilemma	.046	.131	.123	1.04	.81, 1.35
Social capital (solidarity)	.110	.093	1.411	1.12	.93, 1.34
Social capital (trust)	−.080	.103	.602	.92	.75, 1.13
Social capital (social cohesion and inclusion)	.377	.119	10.068**	1.46	1.16, 1.84
RANAS factors					
Vulnerability (personal general risk for diarrhea)	−.626	.113	30.734***	.54	.43, .67
Feeling more respected	−.381	.141	7.327**	.68	.52, .90
Beliefs about costs and benefits (money, space, time)	−1.143	.267	18.246***	.32	.19, .54
Others’ behavior (community)	1.176	.141	69.105***	3.24	2.46, 4.28
Others’ (dis)approval (personally important others’)	.544	.161	11.479**	1.72	1.26, 2.36
Confidence in recovery of broken latrine	.994	.199	25.029***	2.70	1.83, 3.99
Communication	.155	.136	1.297	1.17	.89, 1.52
Constant	−8.13	1.62	25.381***		

*Note. N* = 598. For the overall model of significant context and psychosocial factors (Model 4) R^2^ = .74 (Nagelkerke). X^2^(15) = 468.19, p < .0005. Latrine ownership was coded ‘1’ and no latrine ownership was coded ‘0’.

^a^No relationship as reference category;

OR = odds ratio; CI = confidence interval; **P < .005; ***P < .0005

**Fig. 2:**
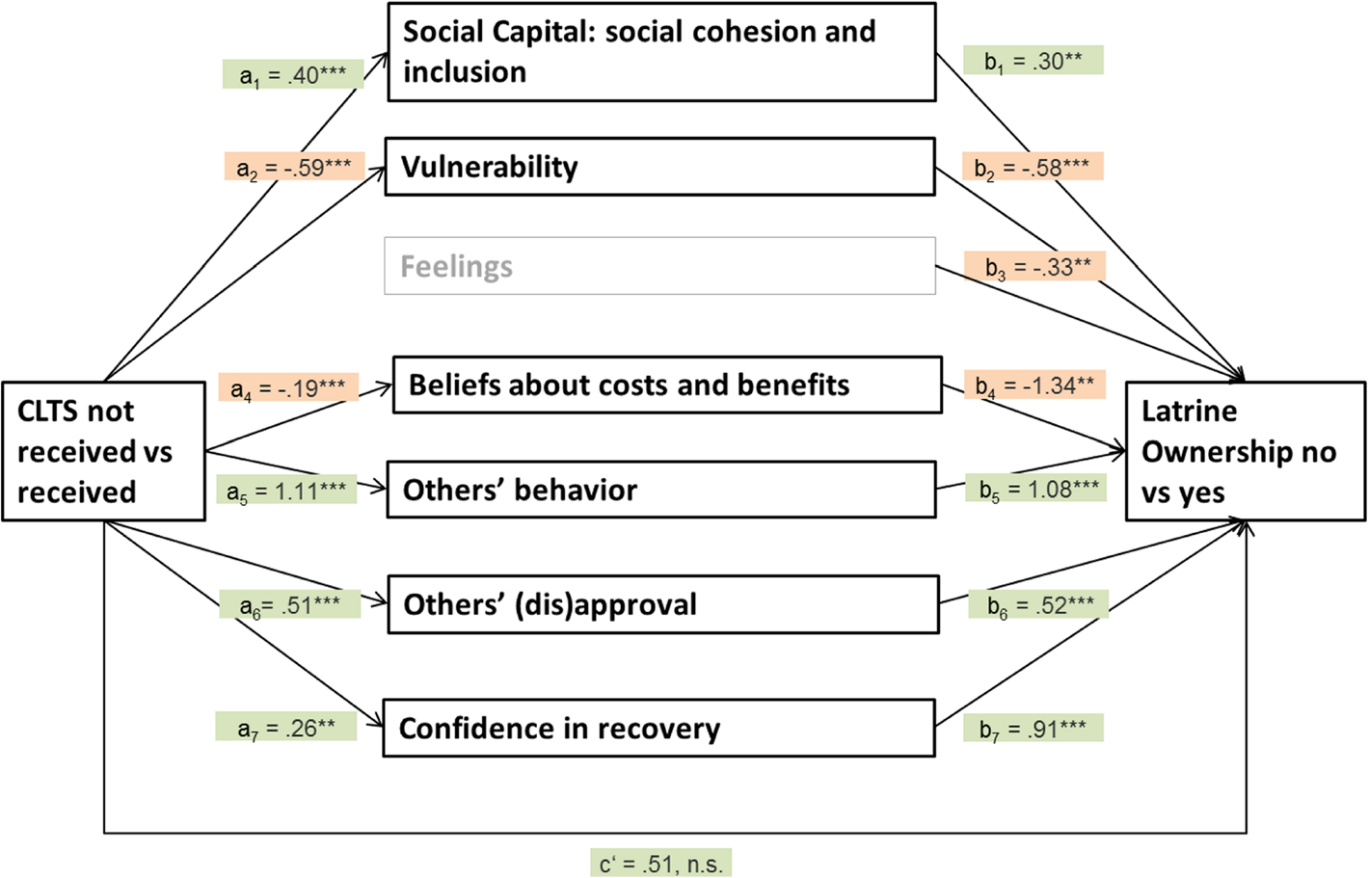
Statistical diagram of the multiple mediation model for the indirect influence of CLTS on latrine ownership through several social and psychosocial context factors. CLTS received was coded ‘1’ and CLTS not received was coded ‘0’. Latrine ownership was coded ‘1’, and no latrine ownership was coded ‘0’. a1 - a7 = unstandardized regression coefficients from linear regressions; b1 – b7 = unstandardized regression coefficients from logistic regression; c’ = indirect effect of CLTS on latrine ownership status

To answer the third research question (Which structural, personal and social context factors mediate the association between CLTS participation and latrine ownership?), social and psychosocial factors identified as relevant in logistic regressions were used as mediators in a multiple mediation analysis. As can be seen in Fig. 2 and Table 4, CLTS indirectly influenced the probability of latrine ownership through its effects on social cohesion and inclusion within the village (OR = 1.127), the personal estimation of vulnerability for diarrhea (OR = 1.409), beliefs about costs and benefits (OR = 1.297), the behavior of community members (OR = 3.327), the (dis)approval of important others of constructing a latrine (OR = 1.309), and the confidence in being able to rebuild a damaged latrine (OR = 1.267). Figure 2 shows that participants who received CLTS felt a stronger sense of cohesion and inclusion within their community (*a*_1_ = .402), felt less vulnerable to becoming infected with diarrhea (*a*_2_ = −.593), estimated the costs for latrine construction as lower (*a*_4_ = −.194), estimated the rate of latrine ownership within their community as higher (*a*_5_ = 1.110), felt stronger approval of important others of constructing a latrine (*a*_6_ = .517), and felt more confident in being able to repair their latrine in case of damage (*a*_7_ = .260). Latrine ownership is associated with a stronger sense of cohesion and inclusion within the community (*b*_1_ = .298), feeling less vulnerable to becoming infected with diarrhea (*b*_2_ = −.578), having lower cost expectations about latrine construction (*b*_4_ = − 1.339), higher estimated rates of latrine ownership within the community (*b*_5_ = 1.083), stronger approval by important others of constructing a latrine (*b*_6_ = .521) and higher confidence in being able to repair a damaged latrine (*b*_7_ = .910). Bias-corrected bootstrap intervals based on 10,000 bootstrap samples and odds ratios were computed for all specific indirect effects (see Table 4). There was no evidence that CLTS is related with the probability of latrine ownership independently of its association with the factors mentioned above (*c’* = .512, *p* = .076).

**Table 4:** Summary of multiple mediation analysis: CLTS indirectly influencing latrine ownership status through its effect on several social and psychosocial factors

Mediator	CLTS			Latrine ownership			Indirect effect (95% CI)			Odds ratio for specific indirect effects (95% CI)
	*B*	*SE*	*p*	*B*	*SE*	*p*	LL	*B*	UL	OR
Social capital	0.402***	0.109	.000	0.298**	0.108	.005	0.028	**0.120**	0.267	1.127
Vulnerability	−.593***	0.115		−0.577***	0.103	.000	0.174	**0.343**	0.555	1.409
Feelings	.026	0.090	.770	−0.327**	0.127	.009	−0.087	− 0.009	0.052	0.99
Beliefs about costs and benefits	−.194***	0.050	.000	−1.339***	0.249	.000	0.113	**0.260**	0.472	1.297
Others’ behavior	1.110***	0.101	.000	1.082***	0.126	.000	0.856	**1.202**	1.582	3.327
Others’ (dis)approval	.517***	0.082	.000	0.521***	0.148	.000	0.096	**0.269**	0.483	1.309
Confidence in recovery of broken latrine	.260**	0.083	.002	0.910***	.177	.000	0.074	**0.237**	0.447	1.267

*Note N* = 593. B = unstandardized regression coefficients from linear regressions (CLTS) and logistic regression (latrine ownership); SE = standard error; CI = confidence interval for specific indirect effects; LL = lower limit; UL = upper limit; OR = odds ratio for specific indirect effects

CLTS received was coded ‘1’, and CLTS not received was coded ‘0’. Latrine ownership was coded ‘1’, and no latrine ownership was coded ‘0’. Bias-corrected bootstrap confidence intervals for the specific indirect effects were computed based on 10,000 bootstrap samples (bold: Significant effects)

## Discussion

The results indicate that building a latrine depends on i) pre-existing social context factors, ii) an intensive social process kicked off by CLTS, iii) CLTS fostering confidence in being able to build and rebuild a latrine, and iv) CLTS communicating the benefits to health of owning a latrine.

It has already been shown that various contextual conditions, such as initial latrine coverage and the difference of facilitating staff, lead to differing levels in the effectiveness of CLTS [6, 20]. Positive social context factors seem to be a prerequisite for a successful CLTS process. The results indicate that a strong sense of cohesion and inclusion, high trust and firm solidarity between the residents, and a high collective ambition to reduce open defecation provide the basis on which CLTS can work successfully. This is implicitly already understood by implementing NGOs, because it is part of the pre-triggering phase, which clarifies how ready a community is for an effective CLTS process. Recent publications stress the need to include further social dynamics analysis in the pre-triggering phase [21, 22]. However, the present study provides clear indications of the social characteristics on which implementation organizations should set their focus: social dilemma, solidarity, trust and social cohesion, and inclusion – all social capital items.

The triggering event of CLTS activates an intensive social process between the members of a community [23–25]. It increases the perception that important others promote latrine building and boosts the attention to latrine building of others in the community, accompanied by strengthening social cohesiveness and inclusion. However, CLTS also raises confidence in being able to build a latrine, by lowering the perceived expenditure involved, and in being able to rebuild a damaged latrine, a point especially relevant in flood-prone areas. In addition, CLTS reinforces the belief that people who own a latrine have improved health. The finding that people with higher education are more likely to own a latrine indicates that CLTS facilitators perhaps should initially appeal to these individuals, as they seem to be more open to latrine building than others. This result supports previous findings by Dickinson and Pattanayak [23] in rural India, which showed that education and income are important predictors of latrine ownership. Shakya et al. [26] showed that the probability of owning a latrine is higher for members of higher castes and for more highly educated individuals.

CLTS seems to cause a snowball effect which leads many community members to construct latrines even if they have not attended the triggering session. This is corroborated by the fact that community members who are not even aware that CLTS has been realized in their community have a higher probability of owning a latrine than households in a community where no CLTS has been conducted. Shakya et al. [26] refer to this effect as contagion, a process of social networks. Our study showed that owning a latrine is highly dependent on the proportion of other social contacts owning a latrine, which suggests that not only information but also behavior change is transferred.

Nearly all factors found to be relevant to latrine ownership in the regression analysis were found to be influenced by CLTS in the mediation analysis. This finding highlights the fact that CLTS addresses all the factors which are relevant for latrine construction, meaning that the CLTS process intuitively affects the factors in the mindsets of individuals that change their behavior with regard to latrine construction.

### How could CLTS be made more effective?

We should first note that CLTS is already quite effective. It addresses the relevant factors in an appropriate way and activates an intensive social process which brings a large majority to construct latrines. This is in line with research that shows CLTS is effective for latrine ownership [6]. However, a more conscious approach to dealing with the important social preconditions in specific communities and a more purposeful tackling of the relevant psychosocial factors could surely improve the effectiveness of CLTS. People who didn’t participate in CLTS, those who feel less connected to their community, those who might be more outsiders, are the ones that need to be focused on and explicitly invited and included in the process. Otherwise for them non-adoption is more probable.

When considering social preconditions in the pre-triggering phase, the implementers of CLTS should take into account proven solidarity, existing trust between the community members, prevailing social cohesion and inclusion, and the existing social cooperation dilemma. The facilitator should seek to determine whether a majority of community members will contribute to a community project and trust that others will also contribute and whether the residents will work together to reach a common goal. Recent publications even stress the importance of including members of every sub-group for an sustainable outcome of CLTS [27]. As individuals that perceive themselves as less connected to their social group are less probable to construct latrines, facilitators should explicitly make sure that members of every sub-group of the community are invited to the CLTS-process equally. Indicative questions for the social preconditions can be found in the Supporting Information of this paper or can be developed for an individual project on request by the authors.

The RANAS model describes in detail how psychosocial factors can be tackled by specific behavior change techniques that are each targeting corresponding factors [28]. To augment the perception of important others’ approval of latrine construction, the support of important community members (chief, health worker, teacher, etc.) should be announced publicly. To improve public notice of a household’s intention to construct a latrine, this intention should be signaled in a public commitment in which the household binds itself and makes this public to everybody. This shows the community how many members have already promised to build a latrine and thus exerts pressure on other members as well as on the household to fulfil its commitment.

In the CLTS triggering session beliefs about costs and benefits could be changed further by emphasizing the low costs and effort of latrine construction.

The session also should refer to the repair of broken latrines. Repairs to broken latrines should be seen as normal and thus should not discourage latrine users. They should be reminded that building a latrine and repairing it requires only moderate effort. The reduction of perceived vulnerability when owning a latrine could be addressed by presenting individual assessments of the risk of diarrhea for each person.

### Limitations of this study

The present study is based on a cross-sectional survey, which does not allow causal directions to be inferred. However, it is the first study to determine how CLTS is related to physical, social, personal, and psychosocial factors and how these in turn are associated with latrine ownership. The study reveals that when CLTS is realized in communities with a positive social context, it is able to initiate an intensive social process based on convictions about health risks, efforts, and confidence in latrine building.

## Conclusions

This study can serve as a model for deciding which factors should be taken into account when planning an improved CLTS intervention; these factors should be measured in a baseline survey to inform the intervention. The same factors should also be measured in an end-line survey to uncover changes in the frequency of latrine construction and in the behavioral factors that were targeted, for example by using the RANAS model of behavior change (example studies are for handwashing [29], cleaning of shared toilets [30], the use of safe wells [31], and the use of safe community water filters [32]).

## Additional files

Additional file 1: Items and Answer Categories for Personal, Physical, and Social Context Factors. (DOCX 16 kb)

Additional file 2: Items and Answer Categories for Psychosocial Factors (RANAS). (DOCX 15 kb)

Additional file 3: Predictors of Latrine Ownership in Logistic Regression Analysis. (DOCX 20 kb)

## Abbreveations

CLTS: Community-Led Total Sanitation
NGO: Non-governmental Organizations
OD: Open Defecation
ODF: Open Defecation Free
OR: Odds Ratio
RANAS: Psychological Model on Behavior Change, containing factors on Risk, Attitudes, Norms, Abilities and Self-Regulation
SCIP: Strengthening Communities through Integrated Programming
WaSH: Water Sanitation and Hygiene
