# Supplementary material for: How does Community-Led Total Sanitation (CLTS) affect latrine ownership? A quantitative case study from Mozambique
Source: BMC Public Health. 2018 Mar 21;18:387. doi: 10.1186/s12889-018-5287-y (PMC5861600; doi:10.1186/s12889-018-5287-y)
Supplement: Supplementary file 1 — Items and Answer Categories for Personal, Physical, and Social Context Factors. (DOCX 16 kb) [file 12889_2018_5287_MOESM1_ESM.docx]

**ADDITIONAL FILE 1**

**Table A1.** Items and Answer Categories for Personal, Physical, and Social Context Factors

| **Factor** | **Item example** | **Answer category** |
| --- | --- | --- |
| **Personal and physical factors** |  |  |
| Age | What is your age? | Open question |
| Relationship status | What is your relationship status? | Open question |
| Years at school | How many years did you go to school? | Open question |
| Able to read/ write | Are you able to read/ write? | Open question |
| Religion | What is your religion? | Open question |
| Household size | How many people live in this household in total? | Open question |
| Household income | What is the average monthly income of your family in Meticais (MZN)? | Open question |
| Risk of flooding | Are any parts of the village subject to flooding? | 1 = not at all subject to flooding to  2= very much subject to flooding |
| Soil conditions | What are the general soil conditions like in the village? | Open question |
| **Social factors** |  |  |
| Social dilemma | How much do you feel your community is working together in reducing open defecation? | 1= not at all to  5= very much |
| Social capital: Solidarity | If a community project does not directly benefit me but has benefits for many others in the community, I would contribute time or money to the project. | 1 = extremely disagree to 7 = extremely agree |
| Social capital: Trust | Most people who live in this community can be trusted. | 1 = extremely disagree to 7 = extremely agree |
| Social Capital: Empowerment and political action | I have the freedom to make important decisions that change my life. | 1 = extremely disagree to 7 = extremely agree |
| Social capital: Collective Action and Cooperation | If there is a sanitation problem in this community, how likely is it that people will cooperate to try to solve the problem? | 1 = not at all likely to 5 = very likely |
| Social Capital: Social cohesion and inclusion | In the last month, how many times have you met with people in a public place either to talk or to have food or drinks? | 1 = never  2 = always |
| Social Identity: In-group Ties | I have a lot in common with other community members. | 1 = extremely disagree to 7 = extremely agree |
| Social Identity: Centrality | In general, being a member of this community is an important part of my self-image. | 1 = extremely disagree to 7 = extremely agree |
| Social Identity: In-group Affects | In general, I’m glad to be a member of this community. | 1 = extremely disagree to 7 = extremely agree |
| Social cohesion: Neighborhood cohesion | I would be willing to work together with others on something to improve my community. | 1 = extremely disagree to 7 = extremely agree |
