# Supplementary material for: How does Community-Led Total Sanitation (CLTS) affect latrine ownership? A quantitative case study from Mozambique
Source: BMC Public Health. 2018 Mar 21;18:387. doi: 10.1186/s12889-018-5287-y (PMC5861600; doi:10.1186/s12889-018-5287-y)
Supplement: Supplementary file 3 — Predictors of Latrine Ownership in Logistic Regression Analysis. (DOCX 20 kb) [file 12889_2018_5287_MOESM3_ESM.docx]

**ADDITIONAL FILE 3**

**Table A3.** Predictors of Latrine Ownership in Logistic Regression Analysis

| Model | B | SE | Wald  X2 (1) | OR | 95% CI |
| --- | --- | --- | --- | --- | --- |
| **Model 1: Personal and physical context factors** |  |  |  |  |  |
| Age | .009 | .008 | 1.29 | 1.01 | .99, 1.02 |
| Relationship status^a^ | .855 | .268 | 10.18** | 2.27 | .25, .72 |
| Years at school | .238 | .064 | 13.75*** | 1.27 | 1.12, 1.44 |
| Ability to read/ write^b^ | .043 | .404 | .01 | 1.04 | .47, 2.30 |
| Religion: Catholic^c^ | -.302 | .208 | 2.11 | .74 | .49, 1.11 |
| Religion: Tribal and other^c^ | -.193 | .376 | .26 | .83 | .40, 1.72 |
| \| Household size \| \| --- \| | .093 | .054 | 2.96 | 1.10 | .99, 1.22 |
| Average monthly income (MZN) of the family | .000 | .000 | 1.51 | 1.00 | 1.00, 1.00 |
| Risk of flooding | -.596 | .090 | 43.51*** | .55 | .46, .66 |
| Soil condition: Clay^d^ | .201 | .221 | .83 | 1.22 | .79, 1.89 |
| Soil condition: Rocky^d^ | -.336 | .365 | .84 | .72 | .35, 1.46 |
| Constant | -.374 | .519 | .52 | .69 |  |
|  |  |  |  |  |  |
| **Model 2: Social context factors** |  |  |  |  |  |
| Social dilemma | ,487 | ,084 | 33,291*** | 1,63 | 1,38, 1,92 |
| Social capital (solidarity) | ,179 | ,058 | 9,441** | 1,20 | 1,07, 1,34 |
| Social capital (trust) | ,204 | ,064 | 10,249** | 1,23 | 1,08, 1,39 |
| Social capital (empowerment and political action) | ,052 | ,071 | ,535 | 1,05 | ,95, 1,21 |
| Social capital (collective action and cooperation) | ,148 | ,106 | 1,972 | 1,16 | ,94, 1,43 |
| Social capital (social cohesion and inclusion) | ,183 | ,073 | 6,174* | 1,20 | 1,04, 1,39 |
| Social identity (in-group ties) | ,071 | ,072 | ,969 | 1,07 | ,93, 1,24 |
| Social identity (centrality) | ,055 | ,093 | ,344 | 1,06 | ,88, 1,27 |
| Social identity (in-group affects) | ,032 | ,071 | ,202 | 1,03 | ,90, 1,19 |
| Social cohesion (neighborhood cohesion | -,097 | ,088 | 1,201 | ,91 | ,76, 1,08 |
| Constant | -4.591 | .845 | 29.531*** | .01 |  |
|  |  |  |  |  |  |
| **Model 3: RANAS factors** |  |  |  |  |  |
| Vulnerability (personal general risk for diarrhea) | -.527 | .104 | 25.471*** | .59 | .48, .72 |
| Vulnerability (general health of community members) | .277 | .188 | 2.177 | 1.32 | .91, 1.91 |
| Vulnerability (defecation-related personal diarrhea risk) | .203 | .292 | .486 | 1.23 | .69, 2.17 |
| Vulnerability (defecation -related diarrhea risk for community members) | -.169 | .291 | .338 | .85 | .48, 1.49 |
| Health Knowledge | -.178 | .196 | .822 | .84 | .57, 1.23 |
| Feelings (proud) | .387 | .262 | 2.181 | 1.47 | .88, 2.46 |
| Feelings (satisfaction) | -.523 | .281 | 3.448 | .59 | .34, 1.03 |
| Feelings (respect) | -.309 | .139 | 4.946* | .73 | .56, .96 |
| Beliefs about costs and benefits (expansiveness) | -.167 | .122 | 1.896 | .85 | .67, 1.07 |
| Beliefs about costs and benefits (money, space, time) | -.931 | .312* | 8.887** | .39 | .21, .73 |
| Others’ behavior (relatives) | .129 | .134 | .930 | 1.14 | .88, 1.48 |
| Others’ behavior (community members) | .996 | .145 | 47.310*** | 2.71 | 2.04, 3.60 |
| Others’ (dis)approval (personally important others) | .564 | .166 | 11.537** | 1.76 | 1.27, 2.43 |
| Others’ (dis)approval (important persons of the community) | -.087 | .137 | .399 | .92 | .70, 1.20 |
| Personal importance | -.033 | .093 | .124 | .97 | .81, 1.16 |
| Confidence in performance | .183 | .136 | 1.818 | 1.20 | .92, 1.57 |
| Confidence in recovery | .706 | .203 | 12.084** | 2.03 | 1.36, 3.02 |
| Confidence in continuation | -.057 | .146 | .152 | .95 | .71, 1.26 |
| How-to-do-knowledge | .251 | .159 | 2.499 | 1.29 | .94, 1.76 |
| Commitment | .156 | .092 | 2.915 | 1.17 | .98, 1.40 |
| Communication | .306 | .120 | 6.488* | 1.36 | 1.07, 1.72 |
| Constant | -7.61 | 1.878 | 16.434*** |  |  |
|  |  |  |  |  |  |
| **Model 4: significant context and RANAS factors from model 1+2+3** |  |  |  |  |  |
| **Context factors** |  |  |  |  |  |
| Relationship status^a^ | .545 | .388 | 1.969 | .58 | .27, 1.24 |
| Years at school | .188 | .070 | 7.247** | 1.21 | 1.05, 1.39 |
| Risk of flooding | -.351 | .128 | 7.546** | .70 | .55, .90 |
| Social dilemma | .046 | .131 | .123 | 1.04 | .81, 1.35 |
| Social capital (solidarity) | .110 | .093 | 1.411 | 1.12 | .93, 1.34 |
| Social capital (trust) | -.080 | .103 | .602 | .92 | .75, 1.13 |
| Social capital (social cohesion and inclusion) | .377 | .119 | 10.068** | 1.46 | 1.16, 1.84 |
| **RANAS factors** |  |  |  |  |  |
| Vulnerability (personal general risk for diarrhea) | -.626 | .113 | 30.734*** | .54 | .43, .67 |
| Feelings (respect) | -.381 | .141 | 7.327** | .68 | .52, .90 |
| Beliefs about costs and benefits (money, space, time) | -1.143 | .267 | 18.246*** | .32 | .19, .54 |
| Others’ behavior (community) | 1.176 | .141 | 69.105*** | 3.24 | 2.46, 4.28 |
| Others’ (dis)approval (personally important others) | .544 | .161 | 11.479** | 1.72 | 1.26, 2.36 |
| Confidence in recovery | .994 | .199 | 25.029*** | 2.70 | 1.83, 3.99 |
| Communication | .155 | .136 | 1.297 | 1.17 | .89, 1.52 |
| Constant | -8.13 | 1.62 | 25.381*** |  |  |

*Note.* *N* = 598. For the model of personal and physical context factors (Model 1) R^2^=.24 (Nagelkerke). X^2^(9) = 107.17, p < .0005. For the model of social context factors (Model 2) R^2^=.26 (Nagelkerke). X^2^(10) = 127.00, p < .0005. For the model of psychosocial factors (Model 3) R^2^=.70 (Nagelkerke). X^2^(10) = 426.85, p < .0005. For the overall model of significant context and psychosocial factors (Model 4) R^2^=.74 (Nagelkerke). X^2^(15) = 468.19, p < .0005. Latrine ownership was coded ‘1’, and no latrine ownership was coded ‘0’.
^a^ no relationship as reference category; ^b^ not able to read/ write as reference category; ^c^ Muslim as reference category; ^d^ sandy as reference category
OR = odds ratio; CI = confidence interval; **P* < .05; ***P* < .005; ****P* < .0005.
